# Integrative personalized medicine care for adjustment disorder of a post-COVID-19 patient: A CARE-compliant case report

**DOI:** 10.1097/MD.0000000000039121

**Published:** 2024-08-02

**Authors:** Won-Kyoung Moon, Ja-Yeon Jeong, Sang-Woo Park, Su-Yeon Yun, Euiju Lee, Seungwon Shin

**Affiliations:** a Department of Sasang Constitutional Medicine, Kyung Hee University Korean Medicine Hospital, Seoul, Republic of Korea; b College of Korean Medicine, Kyung Hee University, Seoul, Republic of Korea; c Department of Sasang Constitutional Medicine, Kyung Hee University College of Korean Medicine, Kyung Hee University Korean Medicine Hospital, Seoul, Republic of Korea; d College of Korean Medicine, Sangji University, Wonju, Republic of Korea.

**Keywords:** acupuncture, adjustment disorders, case reports, herbal medicine, integrative medicine, post-acute COVID-19 syndrome

## Abstract

**Rationale:**

Depression is a common symptom in post-coronavirus disease 2019 (COVID-19) patients, which can be diagnosed with post-COVID-19 depression or adjustment disorder (AD) of post-COVID-19 syndrome. Recently, there have been reports of treating post-COVID-19 syndrome with herbal interventions. However, there are no studies of AD of post-COVID-19 syndrome treated with an integrative approach. This is a CARE-compliant case report of a patient diagnosed with AD of post-COVID-19 syndrome and improved with integrative personalized medicine care (IPMC).

**Patient concerns:**

An 84-year-old female patient presented symptoms of depression, insomnia, palpitations, and dyspepsia after COVID-19 diagnosis.

**Diagnoses:**

The patient was diagnosed with AD due to COVID-19 according to Diagnostic and Statistical Manual of Mental Disorders, Fifth Edition.

**Interventions:**

The patient was treated with the IPMC approach: conventional Western drugs for symptom improvements with herbal medicine, acupuncture, and moxibustion therapies of traditional Korean medicine to enhance her general conditions.

**Outcomes:**

Depression, insomnia, palpitations, dyspepsia, and overall quality of life were assessed through various questionnaires before and after treatment. Scores notably decreased across depression scales, and insomnia severity improved significantly. After treatment, gastrointestinal symptoms vanished, and autonomic nervous system balance improved. Quality of life metrics also showed remarkable enhancement.

**Lessons:**

This study is the first case report to demonstrate improvement in AD of post-COVID-19 symptoms using IPMC. It is noteworthy that the patient in this study tapered off their antidepressant medication after the treatment with the IPMC approach. Further studies are needed to establish more qualified evidence to show the effectiveness and safety of IPMC for AD of post-COVID-19 syndrome.

## 1. Introduction

Post-coronavirus disease 2019 (post-COVID-19) syndrome is a set of new or persistent symptoms lasting 12 weeks or more after the acute phase of severe acute respiratory syndrome coronavirus-2 infection.^[1,2]^ Its symptoms can be categorized into neurological symptoms, such as fatigue and brain fog, and neuropsychiatric symptoms, such as depression and anxiety.^[3]^ Depression is a common symptom in 30% to 40% of COVID-19 patients,^[4–7]^ which can be diagnosed with post-COVID-19 depression or adjustment disorder (AD) of post-COVID-19 syndrome.^[8]^ AD is a maladaptive response to a psychosocial stressor and it is classified as a mental disorder.^[9]^ Based on the Diagnostic and Statistical Manual of Mental Disorders, Fifth Edition criteria, AD can be diagnosed with depressed mood and/or anxiety, disturbance of emotions and/or conduct, and unspecified.^[9]^

There is currently no standardized treatment for AD. According to a systematic review published in 2018, treatments have primarily involved pharmacological interventions, such as antidepressants and anxiolytics, and psychotherapy, particularly cognitive-behavioral therapy. However, pharmacological treatments have frequently reported gastrointestinal and neurological side effects. While psychotherapy has shown some effectiveness, the overall quality of evidence remains low due to methodological limitations.^[10]^ These have led to exploring various complementary and alternative medical approaches, such as acupuncture and herbal medicine.^[11–13]^

Recently, studies have been reported to treat post-COVID-19 syndrome with herbal interventions.^[11–13]^ A study reviewed late complications of COVID-19 cases managed with herbal medicines.^[14]^ Others reported several cases with post-COVID-19 globus sensation,^[15]^ post-COVID-19 syndrome of Soyang type, which is a constitutional type of Sasang constitutional medicine in Korea,^[16]^ or long COVID complaining of cough and sore throat.^[17]^ However, there are no studies or case reports of AD of post-COVID-19 syndrome. This is a case report of a patient diagnosed with AD of post-COVID-19 syndrome and improved with IPMC which refers to an integrative treatment tailored to the individual characteristics of the patient, rather than a standardized and predefined treatment.

## 2. Case presentation

### 2.1. Patient information

An 84-year-old female patient (159 cm and 62 kg) visited a traditional Korean Medicine (TKM) hospital in Seoul, Republic of Korea, in August 2023. She complained of depression, insomnia, palpitations, and dyspepsia that occurred after severe acute respiratory syndrome coronavirus-2 infection. The patient was initially diagnosed with COVID-19 in May 2023 and had completed 3 doses of COVID-19 mRNA vaccination before the infection. The patient, after the COVID-19 struggle, was having trouble falling asleep (taking 1 to 3 hours) and could sleep for only 3 to 5 hours per day with light sleep and frequent awakenings. She also had eating problems, feeling discomfort and heaviness in the upper abdomen after meals, which started 3 months after the COVID-19 diagnosis. She also had a bloated sensation throughout the upper and lower abdomen, along with frequent belching, trembling, and a burning sensation on an empty stomach. These symptoms led to severe discomfort, and the patient occasionally expressed thoughts of wanting to die particularly when feeling overwhelmed. The patient was admitted to a general hospital to manage those symptoms before coming to the TKM hospital and got a result of normal findings in the esophagogastroduodenoscopy.

The patient underwent a hysterectomy 40 years ago and has been taking antihypertensive drugs for the past 30 years. There were no significant disorders in her family history. She did not smoke or drink alcohol. There were no psychologically stressful events recently.

### 2.2. Diagnostic assessment

On the first day of admission, a complete blood count and electrolyte lab were conducted, revealing an elevation in erythrocyte sedimentation rate (26 mm/h), an increase in creatine kinase (439 IU/L), and an elevated uric acid level (6.1 mg/dL). However, other parameters were within their normal ranges. No abnormalities were found in the electrocardiogram, and cardiac markers (creatine kinase-myocardial band, myoglobin, and troponin-I) were also within normal limits. Additionally, no abnormalities were observed in the chest radiography, leading to the decision not to pursue further cardiac-related investigations. Furthermore, an X-ray test did not show any abnormal findings. Confirmation that there were no structural issues related to the digestive system was obtained from a gastroenterology specialist.

In the Yang-do-rak test, an examination that measures the skin’s electrical activity of the 12 meridians to assess the balance of the autonomic nervous system in TKM, the patients showed a mixture of increased (excitation) and decreased (inhibition) activity in various regions. Yang-do-rak is a TKM test that uses changes in microcurrents to reflect the function of meridians and organs.^[18,19]^ Furthermore, in the heart rate variability (HRV) test, which assesses the balance of the autonomic nervous system through variations in heart rate, the autonomic nervous system activity was measured at 116 points (very poor). HRV was first recognized as a potential indicator of autonomic nervous system abnormalities.^[20]^

Based on a previous report indicating that depressive symptoms appeared in approximately 35% of patients after COVID-19 occurrence,^[8]^ assessment questionnaires for depression and sleep disorders were conducted for the patient. She scored 13 points on the Hamilton Depression Rating Scale (HAM-D) indicating mild depression, 27 points on the Beck Depression Inventory (BDI) indicating moderate depression, 21 points on the Korean version of the Geriatric Depression Scale indicating moderate depression, and 20 points on the Patient Health Questionnaire-9 indicating major depression. The severity of insomnia, as measured by the Insomnia Severity Index (ISI), was 23 points, indicating clinically severe insomnia. Additionally, the patient’s quality of life, assessed with the EuroQol Five-dimension Five-level (EQ-5D-5L) questionnaire, yielded a score of 7, and the EuroQol Visual Analogue Scale (EQ-VAS) score was 50.

The patient said that she usually had no worries and considered herself a comfortable elderly person. She did not have a history of depression. She developed depressive symptoms in response to the identifiable stressor of COVID-19, which occurred within 3 months of the onset. The symptoms were clinically significant and resulted in significant impairment in social, occupational, and other important areas of functioning. The above tests have ruled out any other underlying disorders. These allowed us to diagnose her as AD due to COVID-19, following Diagnostic and Statistical Manual of Mental Disorders, Fifth Edition criteria.^[9]^

### 2.3. Integrative personalized medicine care

The treatment plan was designed with 2 stages: the treatment stage and the management stage. The first stage focused on the current symptoms that the patient was complaining of, including discomfort in the digestive system, anxiety, and depression. Once the treatment stage was completed, the process transitioned to the management stage, which was to manage factors that could potentially trigger a relapse.

During the treatment period, an IPMC approach was applied. IPMC refers to an integrative treatment tailored to the individual characteristics of the patient, rather than a standardized and predefined treatment. In other words, in addition to conventional medical interventions for symptom improvements, a holistic approach incorporating TKM treatments, including herbal medicine and acupuncture, was employed to enhance the patient’s overall condition. The treatment was patient-centered, incorporating a Sasang constitutional medicine approach tailored to the patient’s unique constitution.

**Figure 1.**
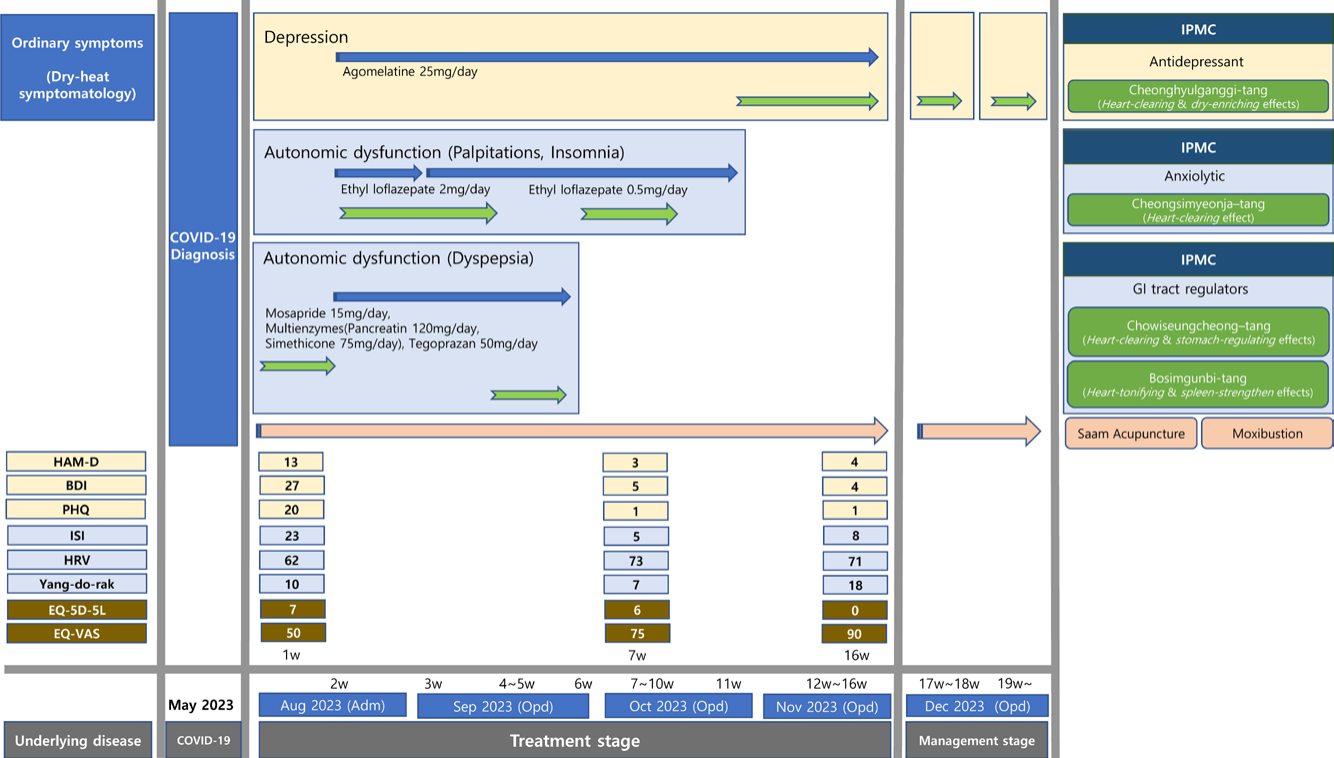
Progress of the patient’s management. Adm = admission; BDI = Beck Depression Inventory; EQ-5D-5L = Euroqol-5 Dimensions-5 Levels; EQ-VAS = Euroqol-Visual Analogue Scale; HAM-D = Hamilton Depression Rating Scale; HRV = heart rate variability; ISI = Insomnia Severity Index; Opd = Outpatient Department; PHQ = Patient Health Questionaire-9.

The patient underwent a 10-day inpatient treatment followed by outpatient treatments 2 to 3 times a week after discharge. All the progress of the patient’s symptoms and treatments are depicted in Figure 1.

#### 2.3.1. Conventional treatment

In collaboration with a psychiatrist for depression management, ethyl loflazepate was administered at 2 mg/day for 1 week, and the dosage was reduced to 0.5 mg/day as the anxiety symptoms improved, continuing for the next 3 weeks. Additionally, agomelatine was prescribed at 25 mg/day for 4 weeks. The patient experienced relief from depressive and insomnia symptoms after 23 days of drug administration, 5 days before completing the 28-day prescription. Although the symptoms disappeared after taking 2 mg/day of ethyl loflazepate for 23 days, the patient continued to take ethyl loflazepate at 0.5 mg/day for an additional 5 weeks and agomelatine at 25 mg/day for ten more weeks for tapering the dosage. Besides, to improve digestive symptoms, 15 mg/day of mosapride, 120 mg/day of pancreatin, 75 mg/day of simethicone, and 50 mg/day of tegoprazan were also prescribed in collaboration with a gastroenterologist.

The patient has been taking clopidogrel (75 mg/day), valsartan (80 mg/day), and hydrochlorothiazide (12.5 mg/day) daily as part of the regular treatment for the underlying condition of hypertension.

#### 2.3.2. Personalized herbal medicine therapy

We adopted 4 therapeutic approaches with herbal medicine during the healing stage*: Stomach-regulating, heart-clearing, heart-tonifying with spleen-strengthening*, and *dry-enriching* effects. In TKM, the *stomach-regulating* effect, meaning the effect to help digestion, focuses on treating gastrointestinal symptoms. The *heart-clearing* effect is aimed at stabilizing overactive and sensitive neurotic symptoms and aiding in sleep, while the *heart-tonifying with spleen-strengthening* effect is a treatment approach for sedation. Lastly, the *dry-enriching* effect, meaning the effect of moistening dryness of the body, targets to prevent constipation, poor circulation, and weakened immune function.^[21]^

Considering the loose and frequent bowel movements in the initial 7 days, we first prescribed Chowiseungcheong-tang (CWSC) for *heart-clearing* and *stomach-regulating* effects. To get her bowel movements back to normal and control her depression, Cheongsimyeonja (CSYJ)-tang for *heart-clearing* effect was administered for 9 days. Subsequently, to help the patient’s digestive function, we administered Bosimgunbi (BSGB)-tang to promote *heart-clearing* and *heart-tonifying with spleen-strengthening* effects for 3 weeks. After the disappearance of all symptoms, Cheongsimyeonja-tang (CSYJ-tang) for 2 weeks followed by Cheonghyulganggi (CHGG)-tang for *heart-clearing* and *dry-enriching* effects were administered for 4 weeks to prevent a recurrence and promote the patient’s health and immune system during the management stage.

All the herbal medicines that were prescribed for the patients are detailed in Table 1.

**Table 1:** Compositions and functions of herbal medicines prescribed for the patient.

Latin name	Botanical name	Latin name	Botanical name	Weight (g/day)	Effect in TKM
*Chowiseungcheong-tang*					
*Lacryma jobi*	Coicis semen	*Castanea crenata*	Castaneae semen	24	*Stomach* *-regulating*
*Plarycodon grandiflorum*	Platycodi radix	*Ephedra sinica*	Ephedrae herba	8	
*Schisandra chinensis*	Schizandrae fructus			8	
*Raphanus sativus*	Raphani semen			12	*Heart-clearing*
*Dimocarpus longan*	Longanae arillus	*Liriope platyphylla*	Liriopes radix	8	
*Acorus gramineus*	Acori graminei rhizoma	*Zizyphus jujuba*	Zizyphi spinosae semen	8	
*Polygala tenuifolia*	Polygalae radix	*Asparagus cochinchinensis*	Asparagi radix	8	
*Bosimgunbi-tang*					
*Cyperus rotundus*	Cyperi rhizoma			18	*Heart-tonifying*
*Zizyphus jujuba*	Zizyphi spinosae semen			16	
*Zingiber officinale*	Zingiberis rhizoma recens			12	
*Cnidium officinale*	Cnidii rhizoma	*Glycyrrhiza uralensis*	Glycyrrhizae radix	6	
*Dioscorea septemloba*	Dioscorae rhizoma			12	*Spleen-strengthening*
*Hordeum vulgare*	Hordei fructus germinatus			10	
*Fraxinus rhynchophylla*	Citri pericarpium	*Pinellia ternata*	Dinelliae tuber	6	
*Phyllostachyos caulis*	Phyllostachyos caulis	*Poncirus trifoliata*	Ponciri fructus	6	
*Atractylodes japonica*	Atractylodis rhizoma	*Atractylodes japonica*	Atractylodis rhizoma alba	6	
*Poria cocos*	Poria sclerotium	*Officinal magnolia*	Machili cortex	6	
*Agastache rugosa*	Pogostemonis herba	*Amomum villosum*	Amomi semen	6	
*Triticum aestivum*	Massa medicata fermentata			6	
*Saussuerea lappa*	Saussureae radix			4	
*Cheongsimyeonja-tang*					
*Nelumbo nucifera*	Nelumbinins semen	*Dioscorea septemloba*	Dioscorae rhizoma	16	*Heart-clearing*
*Asparagus cochinchinensis*	Asparagi radix	*Liriope platyphylla*	Liriopes radix	8	
*Polygala tenuifolia*	Polygalae radix	*Acorus gramineus*	Acori graminei rhizoma	8	
*Zizyphus jujuba*	Zizyphi spinosae semen	*Dimocarpus longan*	Longanae arillus	8	
*Thuja orientalis*	Biotae semen	*Scutellaria baicalensis*	Scutellariae radix	8	
*Raphanus sativus*	Raphani semen			8	
*Dendranthema indicum*	Chysanthemi flos			2	
*Cheonghyulganggi-tang*					
*Scutellaria baicalensis*	Scutellariae radix	*Nelumbo nucifera*	Nelumbinins semen	3	*Heart-clearing*
*Dioscorea septemloba*	Dioscorae rhizoma			3	
*Raphanus sativus*	Raphani semen	*Liriope platyphylla*	Liriopes radix	1.5	
*Asparagus cochinchinensis*	Asparagi radix	*Polygala tenuifolia*	Polygalae radix	1.5	
*Acorus gramineus*	Acori graminei rhizoma	*Zizyphus jujuba*	Zizyphi spinosae semen	1.5	
*Dimocarpus longan*	Longanae arillus			1.5	
*Thuja orientalis*	Biotae semen	*Dendranthema indicum*	Chysanthemi flos	0.75	
*Pueraria lobate*	Pueraria root			6	*Dry-enriching*
*Angelica tenuissima*	Ligustici Tenuissimi rhizoma et radix			3	
*Plarycodon grandiflorum*	Platycodi radix	*Cimicifuga biternata*	Cimicifugae rhizoma	1.5	
*Angelica dahurica*	Angelicae dahuricae radix	*Rheum palmatum*	Rhei radix et rhizoma	1.5	

TKM: Traditional Korean Medicine.

#### 2.3.3. Personalized acupuncture and moxibustion therapies

We have chosen an acupuncture regimen based on the traditional acupuncture theory of meridians. Applying the Saam acupuncture method of TKM, we conducted acupuncture therapies on ST36, LU11, SI5, LI5, LU8, LR4, HT8, and LR2 (bilateral sides). Following the general meridian theory, we applied acupuncture on GB20 for general condition improvement and on ST25, and CV4 for digestive symptom improvement. A total of 21 acupuncture points were used per session. The patient received a total of 18 sessions of acupuncture treatments, 20 minutes each, during the initial 10-day hospitalization (daily) and after discharge (once per 2–3 days during outpatient visits). All acupuncture procedures were performed by a skilled TKM doctor using single-use acupuncture needles (Dongbang Acupuncture Inc., Korea, 0.20 mm × 30 mm stainless steel) inserted to a depth of 5 to 20 mm. The needles were retained for 20 minutes with electrical stimulation at 2 Hz intensity (only for GB20 acupoints). Infrared therapy was administered to the abdominal area using an infrared lamp (Daekyung Electronics, Korea, INFRALUX-300). Detailed information on acupuncture therapy is summarized in Table 2, following STRICTA guideline.

**Table 2:** Acupuncture therapy administered for the post-COVID-19 patient.

1. *Details of needling*
(1) Number of needle insertions per subject per session: 20. (2) Based on liver-sedation theory of Saam acupuncture, we adjusted the acupuncture treatment by adding or subtracting specific acupoints according to the severity of the major issues, namely digestive disorders, anxiety disorders, and depressive disorders. Names of acupoints used: Gyeonggeo (LU8), Joongbong (LR4), Haenggan (LR2), Sobu (HT8), Joksamni (ST36), Sosang (LU11), Yanggok (SI5), Yanggye (LI5), Cheonchu (ST25) Gwanwon (CV4) Poongji (GB20) on both side of the subjects. (3) Depth of insertion: 5–20 mm. (4) Response sought: Simple insertion. (5) Needle stimulation: Manual. (6) Needle retention time: 20 minutes. (7) Needle type: single-use acupuncture needles (0.20 mm × 30 mm stainless steel).
2. *Treatment regimen*
(1) Number of treatment sessions: 18. (2) Frequency and duration of treatment sessions: Once a day for the first 10 days, once per 2 to 3 days afterwards.
3. *Other components of treatment*
(1) Details of other interventions administered to the acupuncture treatment: Electrical stimulation at 2 Hz to both GB20 acupoints for 20 minutes. Moxibustion therapy was performed on CV12 and both ST25 acupoints. (2) Setting and context of treatment: Kyung Hee University Korean Medicine Hospital.
4. *Practitioner background*
(1) Description of participating acupuncturists: a TKM licensed doctor.
5. *Control or comparator interventions*
Not applicable.

Alongside acupuncture, moxibustion therapy involving the application of mugwort fibers heated with fire was performed on CV12 and both ST25, providing a warm moxibustion treatment, also known as meridian heat therapy.

## 3. Results

Symptoms assessment was done with HAM-D, BDI, and Patient Health Questionaire for depression, as well as ISI for insomnia. Assessment for palpitations was done through HRV and Yang-do-rak tests. The overall quality of life due to depression, insomnia, palpitations, and dyspepsia was assessed with EQ-5D-5L and EQ-VAS. All assessments were performed at week 1, week 7, and week 16 from the start of treatment.

HAM-D questionnaire scores decreased from 13 (mild, week 1) to 3 (absence of depression, week 7). BDI questionnaire scores decreased from 27 (moderate, week 1) to 5 (absence of depression, week 7), and decreased from 5 to 4 (absence of depression, week 16). Patient Health Questionnaire-9 questionnaire scores decreased from 20 (major, week 1) to 1 (absence of depression, week 7 and week 16). The ISI questionnaire scores improved from 23 (severe, week 1) to 5 (no clinically significant insomnia, week 7) after treatment. The patient, who initially reported taking 1 to 3 hours to fall asleep, waking 5 to 6 times during the night with shallow sleep, achieved 8 hours of restful sleep on the day of taking CSYJ-tang. Even at week 16, the patient maintained a good sleep state. Palpitations also disappeared with the improvement of depressive symptoms.

After treatment, the patient reported the disappearance of post-meal discomfort and overall upper gastrointestinal symptoms. The patient expressed, “digestion is good, and the stomach feels comfortable.”

The results of the Yang-do-rak test to assess the balance of the autonomic nervous system showed a mix of hyperactivity and hypoactivity before treatment. After treatment, the results showed the absence of hyperactivity and hypoactivity. The autonomic nervous system activity score, examined by HRV, improved slightly from 62 (very poor) before treatment on August 14, 2023, to 73 (poor) after treatment on September 11, 2023. In the follow-up examination conducted on November 7, 2023, the HRV score remained at 71 (poor), indicating that the levels of autonomic nervous system activity were maintained.

One month after treatment, the patient’s quality of life was reassessed. The EQ-5D-5L scores improved from 7 (week 1) to 0 (week 16), and EQ-VAS scores increased from 50 (week 1) to 90 (week 16). When the patient initially visited, she expressed a desire to die. However, by the 16th week of treatment, the patient stated, “I’m living comfortably these days.” This change in expression suggests a positive shift in the patient’s well-being and mental state.

## 4. Discussion

This is a case report of an 84-year-old female patient who recovered from AD of post-COVID-19 syndrome with IPMC. The IPMC included the conventional therapy of Western medicine and the traditional therapy, such as herbal medicine, acupuncture, and moxibustion. It is important that IPMC was applied with 2 stages: the treatment and management stages. The overall symptoms of the patient were improved, measured with several validated questionnaires with IPMC.

AD is recognized as a stress-response syndrome, defined as a maladaptive response to a recognizable stressor, and occurring within 1 month of exposure to the stressor.^[22]^ Recently, there have been reports of AD addressing worrying, insomnia, low mood, and general weakness following the COVID-19 occurrence.^[23]^ Unlike post-traumatic stress disorder and autism spectrum disorder, which are triggered by traumatic events, AD is not restricted to a single source of stress.^[24]^ However, it is often caused by exposure to actual or threatened death, as well as nontraumatic stressful events such as interpersonal conflict, death of a loved one, unemployment, financial difficulties, or illness of a loved one or oneself.^[25]^ In this patient’s case, the COVID-19 outbreak may have been a stressful event. Symptoms of AD are characterized by stress responses that go beyond socially and culturally expected responses to stress and result in deficits in daily functioning.^[24]^ It commonly begins with symptoms such as insomnia and anxiety and is often accompanied by physical symptoms such as dyspepsia and headache. This patient did not have a history of depression or other psychiatric illnesses before the COVID-19 infection and met the diagnostic criteria for AD,^[22]^ as she complained of depression, palpitations, insomnia, and severe dyspepsia since the infection, with a low-quality of life (EQ-5D-5L score of 7, EQ-VAS score of 50) and an inability to function in daily life with the expression “I would rather die.”

Recently, the stress-response syndrome has been proposed as a theoretical concept of AD,^[13]^ using the “mental flu” analogy to explain that not everyone exposed to the same stress will develop an AD.^[26]^ Psychological and pharmacological interventions have been used to treat AD, but the quality of evidence is low.^[10]^ There are no traditional medicine guidelines for the treatment of AD, but according to the guidelines for depression or anxiety, treatment with personalized herbal medicines was suggested with Guibi-tang, Danchi-soyosan, and Shihogayonggolmoryo-tang.^[27]^

The therapeutic principle of CSYJ-tang in TKM is *heart-clearing*, which is focused on treating psychological symptoms of AD.^[21]^ The therapeutic principles of CWSC are *heart-clearing* and *stomach-regulating*, the *stomach-regulating* effect is focused on treating digestive problems. The herbs such as *Lacryma-jobi* and *Castaneae crenata* of CWSC are known to produce the *stomach-regulating* effect in TKM.^[21]^ The therapeutic principles of Bosimgunbi-tang are *heart-clearing* and *heart-tonifying with spleen-strengthening*. The *heart-tonifying with spleen-strengthening* effect focuses on treating mental factors such as insomnia and stress. The herbs such as *Cyperus rotundus* and *Zizyphus jujube* of Bosimgunbi-tang are known to produce the *heart-tonifying with spleen-strengthening* effect.^[21]^ Cheonghyulganggi-tang is administered for *heart-clearing* and *dry-enriching* effects. Herbs such as *Pueraria lobota* and *Plarycodon grandiflorum* are known to produce the *dry-enriching* effects.^[21]^

CSYS-tang is an herbal medicine used for anxiety and depression.^[28]^ It has antioxidant, immune-boosting, anti-inflammatory, and anti-allergic effects, including NF-kB regulation and cytokine modulation.^[29–31]^ Additionally, *Dimocarpus longan*, a constituent of CSYS-tang, has been reported to exhibit antioxidant effects in nonclinical studies.^[32]^ Moreover, it demonstrates antidepressant effects by increasing neurotransmitters in the hippocampus and prefrontal cortex within a chronic mild stress model.^[33,34]^ *D longan* is traditionally used for insomnia, anxiety, and postmenopausal depression,^[35]^ and has been reported to inhibit the decline in myocardial contractile function,^[36]^ and to have antiarrhythmic effects^[37]^ in association with patient-reported palpitations. Another component, *Scutellaria baicalensis*, has been reported to have anti-inflammatory effects.^[38]^

Saam acupuncture, a unique acupuncture treatment in TKM, has immunomodulatory and autonomic modulatory effects.^[39,40]^ Also, ST36 has been used in previous cases of post-COVID-19 syndrome.^[11,12]^ The acupoints of ST36 and GB20 are known to have anti-inflammatory effects, too.^[41,42]^

It is noteworthy that the patient in this study tapered off their antidepressant medication after only 1 week of the IPMC approach. According to the standards for reporting of diagnostic accuracy studies (STARD) in evidence-based depression clinical practice guidelines for primary care, it takes about 3 months to achieve remission after starting an antidepressant, either by increasing the existing medication or adding a new medication.^[43]^ According to current guidelines for the pharmacotherapy of major depressive disorder, the dose adjustment period is 2 weeks after the first dose and should be continued for 6 months up to 2 years to prevent relapse.^[44]^ However, ethyl loflazepate, a benzodiazepine antidepressant used for anxiety and depressive insomnia,^[45]^ has relatively few side effects of psychiatric disorders such as drowsiness and reduced cognition and concentration.^[46]^ This case demonstrated the beneficial effect of integrative and personalized treatment to shorten the administration duration and dose of conventional antidepressant medications.

The treatment plan was designed with 2 stages: the treatment stage and the management stage. The first stage focused on the current symptoms that the patient was complaining of, including depression, palpitations, insomnia, and dyspepsia. During the treatment period, an IPMC approach was applied. IPMC refers to an integrative treatment tailored to the individual characteristics of the patient, rather than a standardized and predefined treatment. In other words, in addition to conventional medical interventions for symptom improvements, a holistic approach incorporating TKM treatments, including herbal medicine and acupuncture, was employed to enhance the patient’s overall condition. The conventional treatment provided standard treatment for the symptoms, while the personalized TKM treatment considering the patient’s constitutional peculiarities helped a shorter treatment period, a reduction in the dose of medication that can cause side effects in long-term use, an increase in treatment effect, a reduction in relapse risk, an improvement in quality of life, and an increase in patient satisfaction. During the management stage, the treatment was focused on managing factors that could potentially trigger a relapse considering the personalized condition. All patients have “ordinary symptoms,” meaning physiological and pathological symptoms that occur frequently and normally due to differences in Sasang constitutions.^[21]^ The treatment was patient-centered, incorporating a constitutional medicine approach tailored to the patient’s unique ordinary symptoms, which was the basis for selecting herbal medicines for the patient.^[21]^

This study is also meaningful because it shows improvement in post-COVID-19 symptoms with validated measures. Standardized and objective symptom questionnaires were used to measure the changes in subjective symptoms. In addition, esophagogastroduodenoscopy, electrocardiogram, and other laboratory tests were performed in collaboration with doctors of Western medicine to exclude underlying causes.

However, this study also has limitations. Polysomnography, thyroid test, and vitamin B12 test were not performed. Although there is no evidence that laboratory tests are helpful in the diagnosis of AD, thyroid and vitamin B12 tests may help identify temperamental causes of depression.^[44]^ Also, it is not deniable that the IPMC approach lacks evidence. Therefore, the effectiveness and safety of each intervention (herbal medicine, acupuncture, or moxibustion) for AD of post-COVID-19 syndrome should be explored more in the future. Despite the use of validated questionnaires, HRV, and the Yang-do-rak tests, it might not be enough to rely on the patient’s subjective symptoms for the effect assessment. Furthermore, the naturally improving progress of the symptoms could not be ruled out.^[47]^ Therefore, further studies of IPMC for AD of post-COVID-19 syndrome are needed.

Integrating herbal medicine with conventional treatments requires careful consideration of potential interactions. A study evaluating the combination of herbal medicine and conventional therapies for COVID-19 found that herbal medicine significantly reduced clinical symptoms and improved recovery outcomes with mild adverse events.^[48]^ Another review of herbal medicines for the treatment of depression underscores the importance of understanding the interactions between herbal supplements and antidepressants.^[49]^ Yet there are no preceding studies on the interactions between the specific herbal and conventional medicines used in this case. The Ministry of Health and Welfare in Korea is increasing support for scientific investigation into herbal-conventional medicine interactions to ensure safer integrative healthcare services.^[50]^ Until scientific evidence is available, it is necessary to consider the potential and unexpected interactions and adverse effects. It might be helpful to closely monitor liver and kidney function during collaborative treatment.

## Author contributions

**Conceptualization:** Euiju Lee.

**Funding acquisition:** Euiju Lee.

**Investigation:** Won-Kyoung Moon, Ja-Yeon Jeong, Sang-Woo Park, Su-Yeon Yun, Euiju Lee, Seungwon Shin.

**Methodology:** Euiju Lee, Seungwon Shin.

**Project administration:** Euiju Lee.

**Visualization:** Won-Kyoung Moon, Ja-Yeon Jeong, Sang-Woo Park, Su-Yeon Yun.

**Writing – original draft:** Won-Kyoung Moon, Ja-Yeon Jeong, Sang-Woo Park, Su-Yeon Yun.

**Writing – review & editing:** Euiju Lee, Seungwon Shin.
